# SMART Syndrome: Case Report and Review of the Literature

**DOI:** 10.5334/jbsr.3198

**Published:** 2023-07-06

**Authors:** Christophe Dossin, Dario Di Perri, Nicolas Whenham, Anna Paola Bocchio, Idil Gunes Tatar

**Affiliations:** 1 Cliniques universitaires Saint Luc, Department of radiology, BE; 2 Cliniques universitaires Saint Luc, Department of radiotherapy, BE; 3 Cliniques universitaires Saint Luc, Department of radiation oncology, BE

**Keywords:** Radiation, Stroke-like, Migraine, SMART

## Abstract

Stroke-like migraine attacks after radiation therapy (SMART) syndrome is a rare condition characterized by stroke-like deficits, seizures, and headache that can occur years after radiation therapy (RT) to the brain. RT is a cornerstone in the treatment of primary brain tumours and is indicated in more than 90% of patients. It is therefore essential to be aware of this entity to prevent misdiagnosis leading to inappropriate treatment.

In this article, typical imaging findings of this condition are presented through a case report and review of the literature.

## Introduction

First described in 1995 by Shuper et al., the SMART (Stroke-like migraine attacks after radiation therapy) syndrome is a delayed complication of brain irradiation with a typical presentation. Symptoms consist of stroke-like deficits such as aphasia, negligence, hemianopsia, epileptic seizures, and migraine, which appear years after RT (1–37 years) [1,2]. The physiopathology is unclear, but it is thought to be related to vascular dysfunction due to delayed radiation-induced neurotoxicity [3]. RT is a cornerstone in the treatment of primary malignant brain tumours and is indicated in more than 90% of the patients [4]. It is therefore essential to be aware of this entity to prevent misdiagnosis leading to inappropriate treatment.

Herein we report a case of a 61-year-old female patient with SMART syndrome presenting 10 years after RT for a grade 4 astrocytoma.

## Case History

A 61-year-old female patient with a history of left frontal isocitrate dehydrogenase (IDH) – mutant astrocytoma (grade 4) presented to the emergency department with headache, right upper limb paresthesia and aphasia.

Ten years earlier, the patient was treated with macroscopic complete resection followed by chemoradiation (60 Gy in 30 fractions with concurrent and adjuvant temozolomide). The patient subsequently underwent regular follow-up with magnetic resonance imaging (MRI) showing an absence of recurrence. The last MRI was performed two months before her presentation to the emergency department.

**Figure 1:**
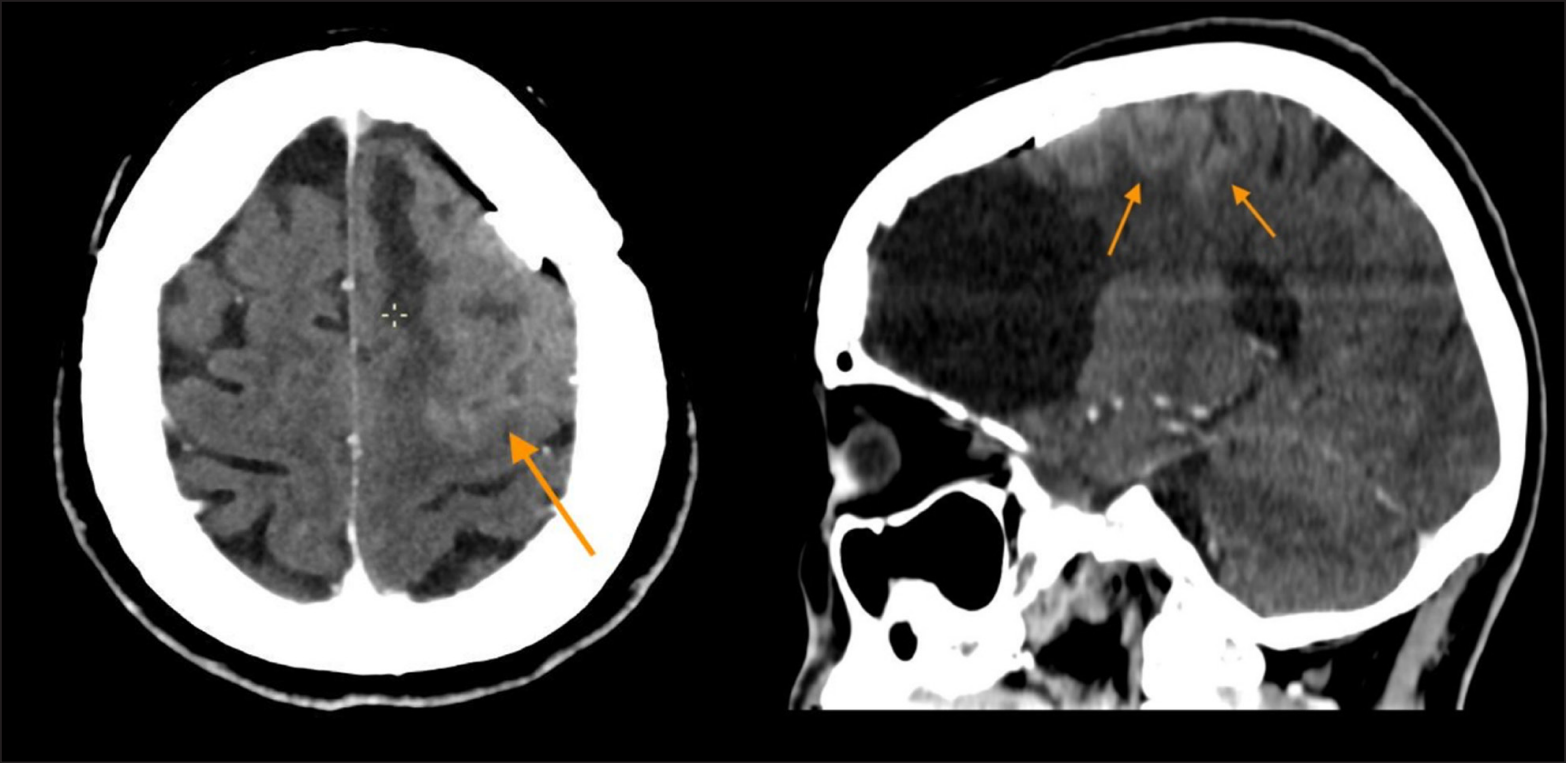
Axial and sagittal CT section shows corticopial enhancement in the left posterior frontal lobe surrounding the resection cavity.

**Figure 2:**
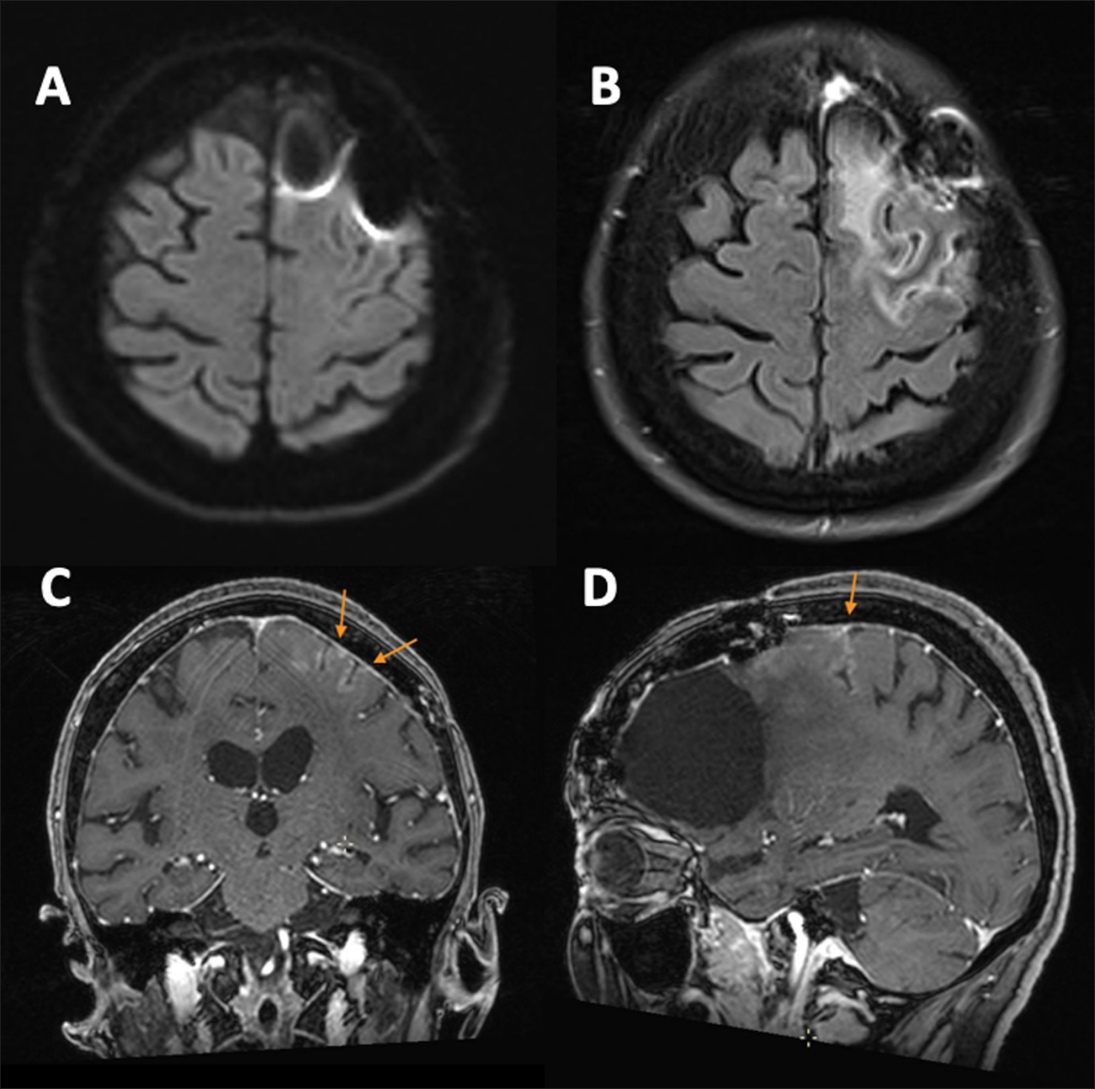
Brain MRI at presentation with no evidence of restriction of diffusion **(A).** Marked left posterior frontal gyral edema on axial FLAIR **(B)** associated with cortical gyriform gadolinium enhancement on coronal and sagittal T1-weighted **(C–D)**.

**Figure 3:**
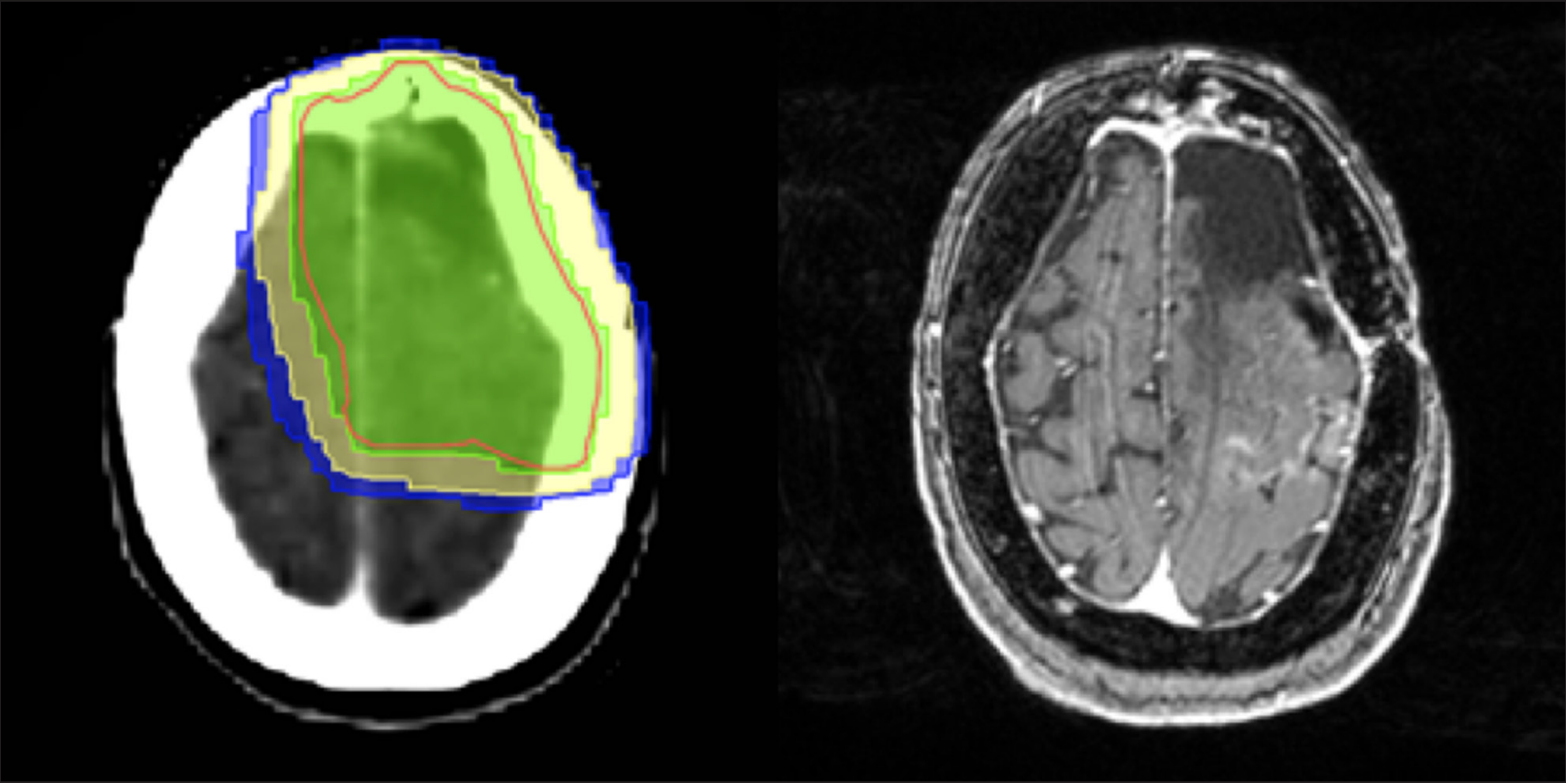
Radiotherapy dose distribution with correlating axial post-contrast T1-weighted image demonstrating the cortical enhancement at presentation.

As part of the diagnostic workup of the patient, a cerebral computed tomography (CT) scan was performed which demonstrated corticopial enhancement in the left frontal lobe located posteriorly to the resection cavity (Figure 1). An additional magnetic resonance imaging (MRI) examination was performed the next day which demonstrated slight edema of the left frontal gyri, hyperintensity on T2/FLAIR imaging, persistence of the corticopial enhancement without restricted diffusion or significant hyperperfusion (Figure 2). Lack of diffusion restriction ruled out the preliminary diagnosis of stroke. A differential diagnosis was then evoked between a tumoral recurrence in the form of leptomeningeal carcinomatosis and an infection. Since the enhancement pattern was not nodular and hyperperfusion was not present, recurrence of the tumor was ruled out. Taking into account the focal involvement of a limited part of the frontal lobe and absence of clinico-biological correlation (negative cerebral fluid for infectious conditions as well as for the neoplastic cells), infection was also eliminated. The previous history of RT to this area of the brain (Figure 3) and the stroke like presentation combined with the presence of headache were compatible with the diagnosis of SMART syndrome.

**Figure 4:**
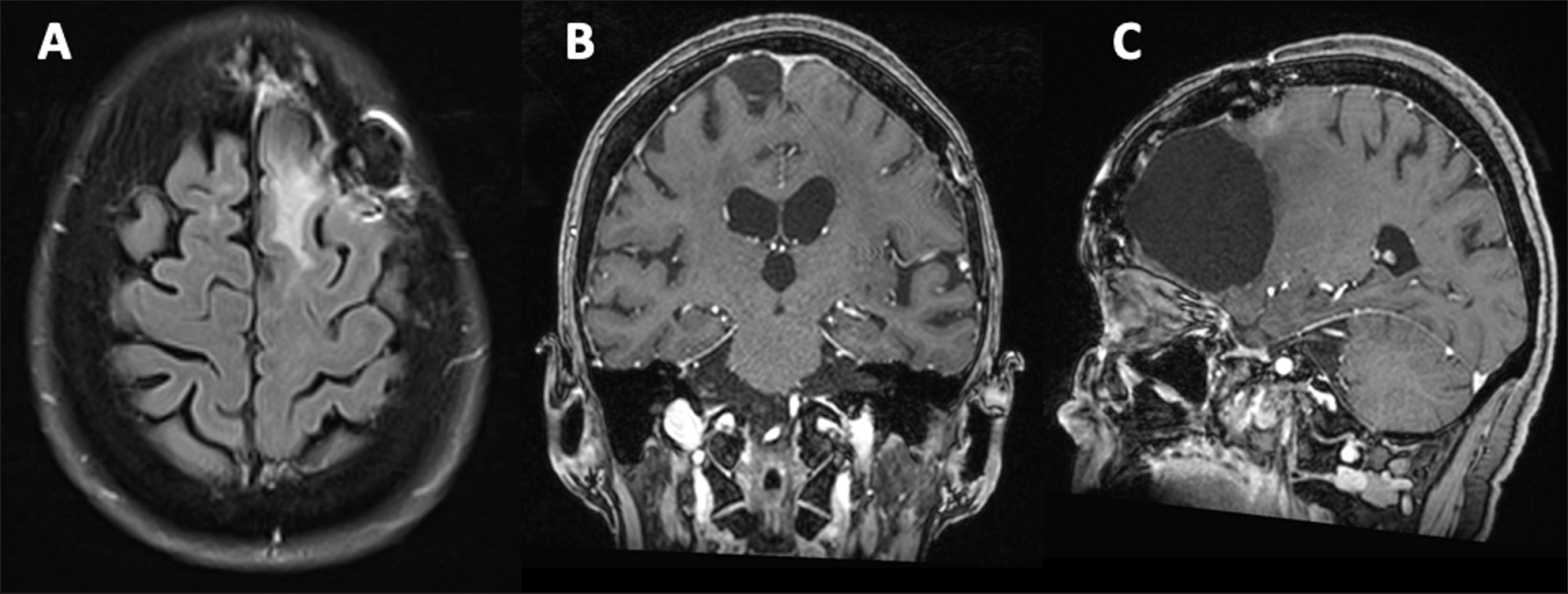
Complete resolution of findings one month later on axial FLAIR **(A)**, coronal and sagittal postcontrast T1-weighted imaging **(B – C)**.

The patient received a loading dose of antiepileptics (1g of Keppra) in the emergency room. No corticosteroids were administered. The patient presented a complete resolution of the neurological symptoms and was discharged from the hospital two days after admission. MRI was repeated one month later and showed complete disappearance of the cortico-meningeal enhancement (Figure 4) and lack of diffusion restriction.

## Comments

The SMART syndrome is a diagnosis of exclusion in patients with a history of prior cranial RT, more frequently in patients who received doses of more than 50 Gy, but it is also known to be associated with lower radiation doses [1,10]. The characteristic imaging findings and the temporal evolution should be known by clinicians and radiologists. Its prompt diagnosis is important to avoid unnecessary invasive investigations which can potentially cause complications related to the procedure, as well as misdiagnosis (e.g., tumour progression), leading to incorrect therapy.

The imaging modality of choice is MRI [3]. The hallmarks of SMART syndrome include a unilateral cortical hyperintensity on T2-weighted and FLAIR imaging, swelling and gyriform cortical enhancement in the areas of the brain that have undergone radiation [1,2,3,5,6,7,8,9,10,11]. The cortical enhancement appears within the first week of symptom onset and generally disappears after one month but in some cases the abnormalities can persist over months [5]. Some notions remain unclear regarding the presence of diffusion restriction and perfusion imaging abnormality. In the majority of cases encountered, the affected area does not show diffusion restriction making the differential diagnosis with acute ischemia easy. However, Singh et al. and Bozkurt et al. reported two cases with diffusion restriction without evidence of a final infarct [6,7]. These patients would be more likely not to recover by ten weeks [7]. In SMART syndrome, no significant increase in cerebral blood flow is found, although some cases have generally reported a transient increase in blood flow at the affected area explained by mild congestion of the overlying pial vessels [1,3,8,9].

The diagnostic criteria for the SMART syndrome can be summarised as:

1. A history of previous focal, cranio-spinal or whole-brain RT.
2. A triad of seizures, headache and hemiparesis which occur years after RT.
3. A gyriform cortical gray matter enhancement and cortical hyperintensity on T2-weighted images and FLAIR.
4. Exclusion of other pathologies like tumor recurrence.

The pathophysiology of the SMART syndrome is yet to be understood. The theories include endothelial damage and vascular dysfunction leading to a failure of autoregulation with neuronal impairment, explained by a reduced threshold value for cortical spreading [2,3].

Although SMART syndrome is considered a benign condition with complete recovery of symptomatology and radiological abnormalities, Black et al. reported some cases with incomplete recovery and persistence of radiological abnormalities such as cortical laminar necrosis [9].

There is still no consensus regarding the treatment, but corticosteroid is the most frequently used drug. According to Jia et al., steroid pulse therapy (1000mg/day, 5 days) would accelerate recovery and provide speedy diagnosis of SMART syndrome [5]. There are other authors who suggest the use of calcium channel blockers such as verapamil in acute-phase treatment considering the to the vascular dysfunction in the pathophysiology of the syndrome [10].

## Conclusion

Recognition of the SMART syndrome by clinicians and radiologists in a patient with a history of brain RT and presenting with stroke-like deficits, seizures, and headache is essential to its prompt management and to avoid invasive diagnostic procedures as well as misdiagnosis leading to inappropriate treatment.
